# Lacrimal Canaliculus Imaging Using Optical Coherence Tomography Dacryography

**DOI:** 10.1038/s41598-018-27802-6

**Published:** 2018-06-28

**Authors:** Masahiro Fujimoto, Akihito Uji, Ken Ogino, Tadamichi Akagi, Nagahisa Yoshimura

**Affiliations:** Department of Ophthalmology and Visual Sciences, Kyoto University Graduate School of Medicine, 54 Shogoin Kawahara-cho, Sakyo-ku, Kyoto 606-8507 Japan

## Abstract

Lacrimal canaliculus (LC) has a key role in tear drainage, but it is difficult to evaluate the LC in detail, using the existing examinations. In this study, our novel LC imaging technique provided the high-resolution images of LC in a non-invasive manner. Three-dimensional images of LC were acquired via the palpebral conjunctiva from 20 healthy volunteers (20 eyes) and 10 patients with various lacrimal disorders (10 eyes), using optical coherence tomography (OCT) dacryography (OCTD). The LC images showed morphological differences between the vertical and horizontal segments. The function of LC could be evaluated by measuring the intralumen signal intensity over time after instillation of a contrast agent (2% rebamipide ophthalmic suspension). OCTD clearly visualised the blind extremity of the LC in four patients with punctal obstruction, which was useful for deciding the punctal incision location. In one patient with canalicular obstruction, contrast agent successfully highlighted the LC that had become narrow toward the site of obstruction. Significant differences were not found in the function and morphology of LC between the patients with NLDO and the healthy subjects. OCTD may be a useful tool for LC imaging, because it facilitates quantitative and simultaneous evaluation of LC morphology and function.

## Introduction

Epiphora is a common ocular complaint that can degrade the vision-related quality of life, particularly during outdoor activities^1^. Woog^2^ reported that the average annual incidence of symptomatic acquired nasolacrimal duct obstruction (NLDO) was 30.47 per 100,000 individuals, with an increase in the incidence during the nearly 25-year study period. Because the number of patients with symptomatic NLDO will continue to increase^2^, further studies for the development of better evaluation and treatment techniques for NLDO are necessary.

The dye disappearance test and probing/irrigation are frequently used for the evaluation of the lacrimal outflow^3–5^. These procedures can be easily performed, but only indirectly examine the lacrimal passage. It can also be imaged using computed tomography dacryocystography and lacrimal scintigraphy^6–10^. However, it is difficult to evaluate the lacrimal canaliculus (LC) in detail using these existing examinations.

In this study, we developed a novel LC imaging technique known as optical coherence tomography dacryography (OCTD), which involves the use of a custom-made swept-source OCT system and a contrast agent, and used it to describe the morphology and function of LC in healthy volunteers and patients with various disorders of the lacrimal drainage system.

## Results

We included 20 eyes of 20 young, healthy Japanese volunteers (10 men, 10 women; average age, 28.3 ± 3.08 years; range, 23–36 years; Table 1) and 10 eyes of 10 patients with lacrimal drainage obstruction (four punctal obstructions, one canalicular obstruction, and five NLDOs; Table 2).

**Table 1:** Demographic and ocular characteristics of healthy volunteers recruited for assessment of the lacrimal canaliculus (LC) using optical coherence tomography dacryography.

n, eyes/subjects	20/20
Age (years)	28.3 ± 3.1
Morphology of the vertical LC	
Anteroposterior lumen length (µm)	356 ± 82
Morphology of the horizontal LC	
Anteroposterior lumen length (µm)	143 ± 23
Transverse lumen length (µm)	2900 ± 421
Lumen area (mm^2^)	0.325 ± 0.114
Epithelium thickness (µm)	89 ± 12
Washout time (min)	
Vertical LC	6.55 ± 1.91
Horizontal LC	6.60 ± 1.93

Data are presented as mean ± standard deviation where applicable.

**Table 2:** Demographics of the patients with lacrimal pathology.

Case	Age (yrs)	Sex	Diagnosis	Past Medical History	History of Lacrimal Surgery
A	58	M	Punctal obstruction	None	None
B	63	F	Punctal obstruction	Allergic conjunctivitis	None
C	68	F	Punctal obstruction	Glaucoma	None
D	72	F	Punctal obstruction	Chronic conjunctivitis	None
E	69	F	Canalicular obstruction	None	None
F	68	M	NLDO	Chronic sinusitis	SI
G	75	M	NLDO	None	None
H	62	F	NLDO	None	None
I	71	F	NLDO	Glaucoma	None
J	83	F	NLDO	Chronic sinusitis	None

NLDO = nasolacrimal duct obstruction.

SI = Silicon intubation.

### Morphological features of the lacrimal canaliculus in the healthy volunteers

LC was successfully imaged with sufficient quality using OCTD in all healthy volunteers. The horizontal and vertical lumen lengths are summarised in Table 1. LC imaging using OCTD enabled noninvasive visualisation of LC from the puncta to the horizontal segment without the use of a contrast agent. After instillation of the rebamipide ophthalmic suspension as a contrast agent, particle light scattering facilitated visualisation of the LC lumen along the entire length of the scan. The LC lumen was identified as a high-intensity band that was easily differentiated from the LC epithelium, which appeared as narrow bands between the Horner’s muscle and the LC lumen.

**Figure 1:**
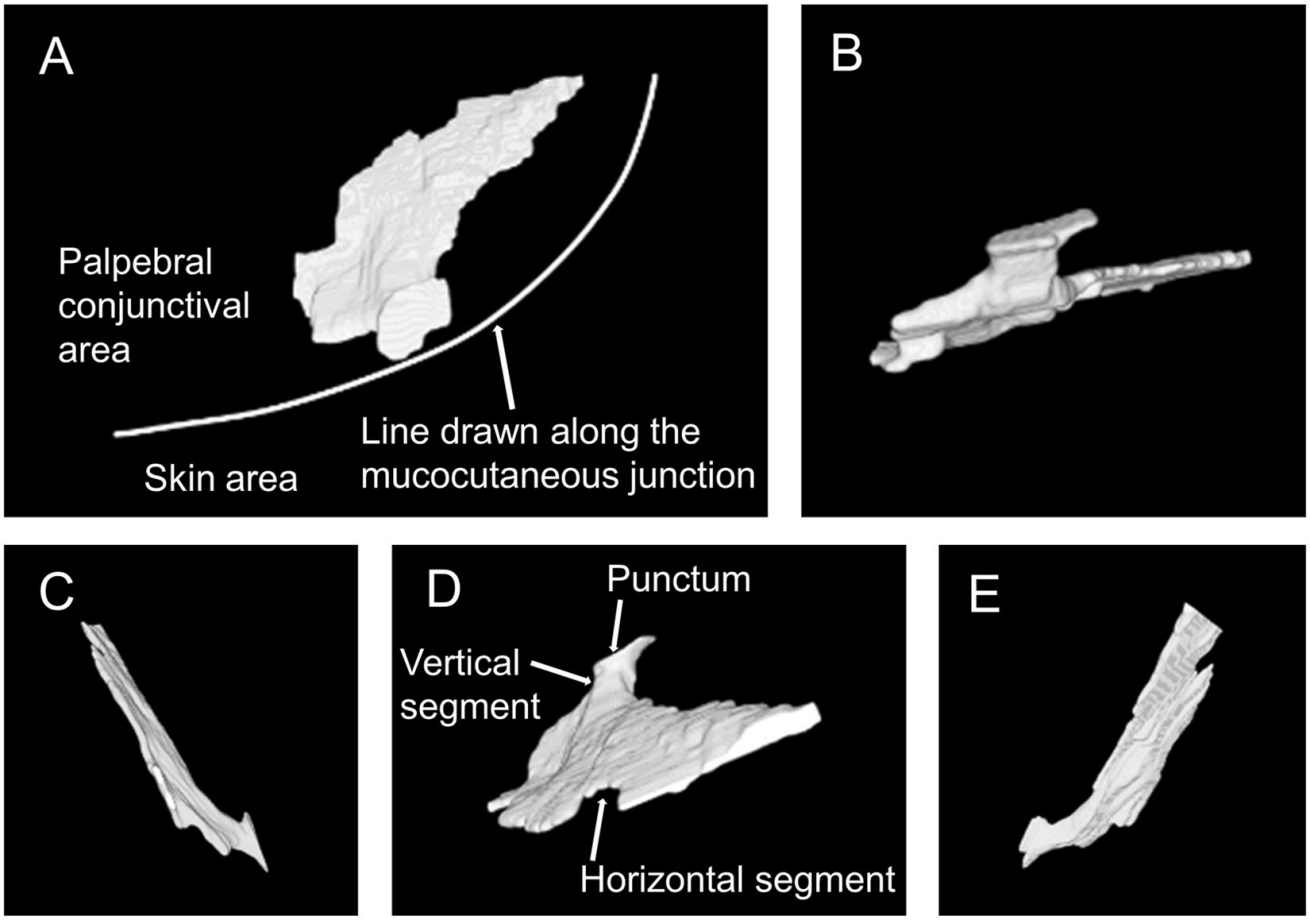
Three-dimensional optical coherence tomography dacryography images of the lumen of the lacrimal canaliculus in a healthy individual. A narrow duct near the punctum spreads out widely toward the distal side. The horizontal segment appears extremely flat, while the vertical segment appears more tubular. Front (**A**), bottom (**B**), temporal (**C**), top (**D**), and nasal (**E**) views can be observed.

Longitudinal OCTD sections obtained along LC showed a wide lumen near the punctum, with the vertical segment exhibiting a flask-like shape. In contrast, the segment distal to the punctum exhibited a narrower and straighter lumen. The anteroposterior lumen length in the vertical segment was 356 ± 82 µm, which was significantly greater than that in the horizontal segment (143 ± 23 µm; *P* < 0.001). Cross-sectional images of the horizontal LC showed a flat lumen with a transverse length of 2900 ± 421 µm, which was significantly greater than the anteroposterior length (143 ± 23 µm; *P* < 0.001). 3D LC reconstructions revealed a narrow duct near the punctum that widely spread out toward the distal side (Fig. 1). There was no significant difference between men and women in morphological measurements.

### Lacrimal canaliculus function in the healthy volunteers

The signal intensity of tears in the lumen increased immediately after rebamipide instillation, peaking at 1 minute after drop administration in both the vertical and horizontal LC segments. After 1 minute, the signal intensity gradually decreased, with a statistically significant change at 3 minutes after rebamipide administration (see Supplementary Fig. S1). The average T1/2 for the vertical and horizontal LC segments was 6.55 ± 1.91 and 6.60 ± 1.93 minutes, respectively, with no significant differences between the men and women (*P* = 0.90).

### Lacrimal canaliculus morphology and function in the patients with lacrimal drainage obstruction

**Figure 2:**
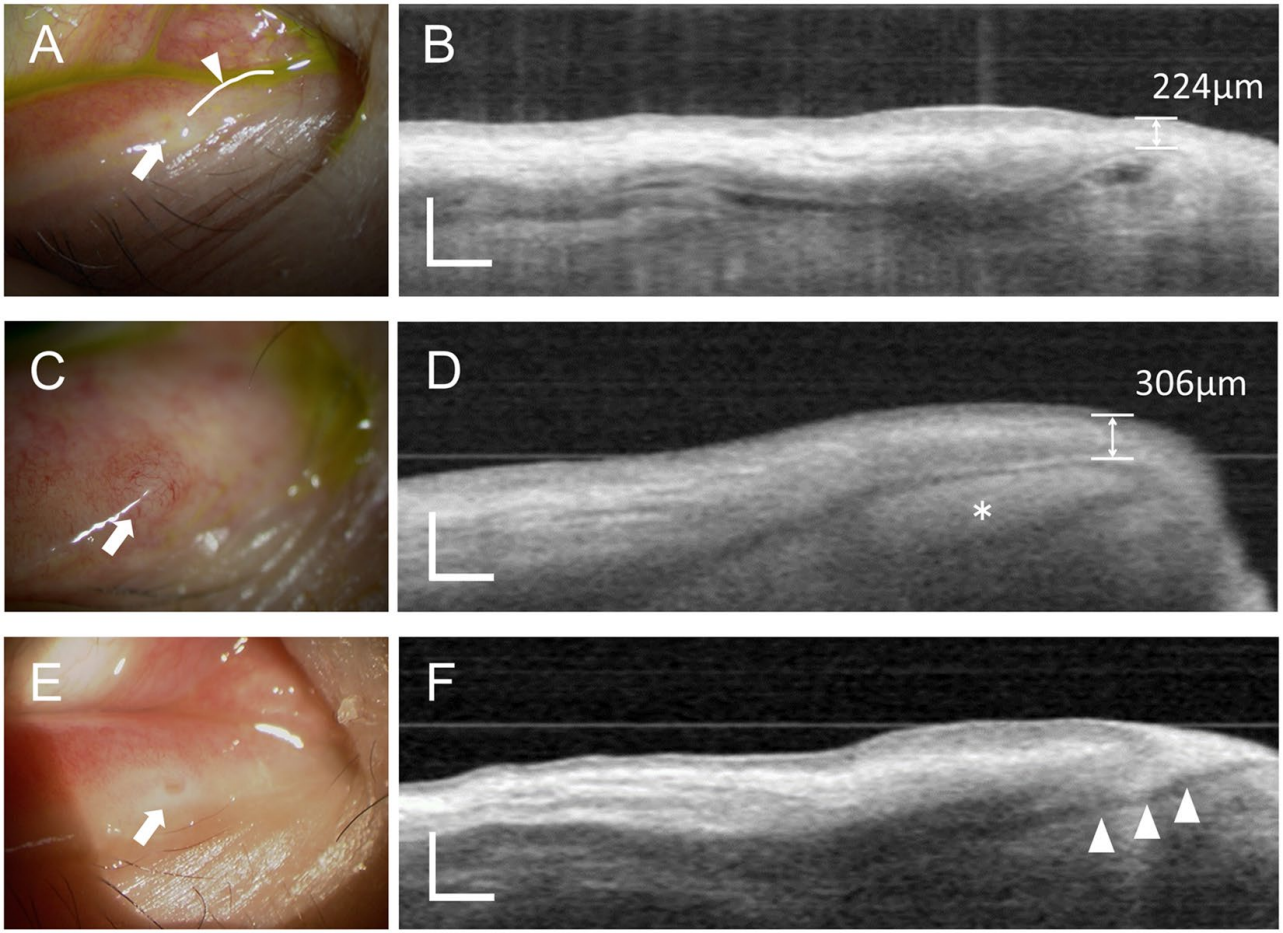
Optical coherence tomography dacryography (OCTD) images of the lacrimal canaliculus (LC) in patients with lacrimal drainage obstruction. (**A**,**B**) Images for a 67-year-old woman with punctal obstruction. (**A**) An image of the right medial lower eyelid. The lacrimal punctum (white arrow) is closed and the lacrimal canaliculus is not clear. (**B**) OCTD B-scan acquired along the curved line in (**A**) (arrowhead). The blind extremity of LC is confirmed at 224 µm under the conjunctival membrane. (**C**,**D**) Images for an 84-year-old woman with punctal obstruction. (**C**) Image of the right medial lower eyelid. The closed punctum (white arrow) is slightly bulging and accompanied by congestion. (**D**) The dilated lumen of the blind extremity of LC accompanied by congestion (asterisk). Dilation of the LC lumen filled with high-intensity material can be observed. OCTD-assisted punctal incision to access the blind extremity resulted in the emission of pus, which corresponded to the high-intensity material on the OCTD images. The average depth of the blind extremity from the conjunctiva was 259 ± 34 μm. (**E**,**F**) Images for a 44-year-old woman with canalicular obstruction. (**E**) Image of the right medial lower eyelid. The lacrimal punctum (white arrow) is confirmed. (**F**) The rebamipide suspension (contrast agent) has entered LC and reveals its obstruction. LC has become narrow toward the site of obstruction. Scale bar = 500 µm.

In all 10 patients, the horizontal LC segment was confirmed to lie beneath the conjunctiva before instillation of the contrast agent. After contrast agent instillation, all patients except the four with punctal obstruction showed an increase in the intensity in the LC lumen. In four patients with punctal obstruction, the blind extremity was identified beneath the conjunctiva (Fig. 2), which was helpful for both diagnosis and treatment; all cases subsequently underwent successful punctal incision using the position and depth of the LC blind extremity as references. The average depth of the blind extremity from the conjunctiva was 259 ± 34 µm. In one patient with punctal obstruction (Table 2, patient D), who had chronic conjunctivitis for at least two years, dilation of the LC lumen filled with high-intensity material was observed, although the irrigation test via the upper punctum was normal. OCTD-assisted punctal incision to access the blind extremity resulted in the emission of pus, which corresponded to the high-intensity material on the OCTD images, and there was no apparent dacryolith in the lacrimal passage. In one patient with canalicular obstruction, contrast agent instillation clearly disclosed the degree and location of the obstruction, which was seen as a filling defect. The flask-like dilation of the vertical segment observed in the healthy volunteers was not detected. There was no apparent abnormality in the LC morphology and function in the five patients with NLDO. The average T1/2 for these patients was 6.80 ± 1.94 for the vertical segment and 7.20 ± 1.33 for the horizontal segment. T1/2 for both the vertical and horizontal segments showed no significant differences between the patients and healthy volunteers (*P* = 0.80 and *P* = 0.54, respectively).

## Discussion

In the present study, we performed OCTD using a prototype OCT system for evaluation of the LC morphology and function in healthy volunteers and patients with various disorders of the lacrimal drainage system. This OCTD system enabled us to obtain high-resolution images of LC *in vivo*, with clear visualisation of LC microstructures such as the epithelium and lumen. These images from dense cube scans allowed the performance of both qualitative and quantitative evaluations because of higher axial and transverse resolutions compared with those provided by other imaging modalities. To the best of our knowledge, this is the first study that used contrast agents to enhance OCT images in humans. Specifically, the contrast agent clearly defined the LC lumen, and changes in the intralumen signal intensity over time were used to quantify the LC discharge function.

The horizontal segment in the OCTD image appeared extremely flat, while the vertical segment exhibited more of a tubular shape. In cross-sectional images of the horizontal segment, the transverse to anteroposterior lumen length ratio was >10, which is considerably larger than the previously reported ratio of approximately 4 (acquired via histological examinations)^11^. The vertical and horizontal segments are anatomically different, with the vertical segment encircled by thick fibrous tissue^12^, and the horizontal segment surrounded by Horner’s muscle^13,14^, which may be affected by eyelid eversion during the examination.

The function of the lacrimal passage is currently evaluated in the clinical setting using lacrimal scintigraphy^6,7,15^, an imaging test that examines the presac, preduct, and intraduct regions^15^. Although OCTD is limited to the presac region (signal attenuation prevents imaging of deeper lacrimal duct structures), T1/2 of tears in LC was measurable and was in good agreement with previous values obtained using lacrimal scintigraphy^16^. Other benefits of OCTD over scintigraphy include better ease of performance and nonrequirement of a radioactive contrast agent. Given the accuracy of OCTD for evaluation of the LC structures and function, we expect this noninvasive imaging technique to be adopted in clinical practice for assessment of the lacrimal outflow status.

The OCTD procedure is supported by three main pillars, including the swept light source, image processing techniques to reslice the cube scan, and image-enhancing contrast agents. The swept light source produces light that penetrates deep into tissue, reslicing of the cube scan allows LC reconstruction and analysis of its complicated 3D structure, and the contrast agent enhances LC images for differentiation between the LC lumen and surrounding tissues. We could also evaluate LC function using the contrast-enhanced OCTD images. Following rebamipide instillation, the boundary between the mucosal membrane and the lumen became evident in the horizontal segment, and this enabled the acquisition of more accurate morphological measurements from each LC segment.

We also performed OCTD imaging for patients with three types of lacrimal drainage obstructions. In all cases, direct visualisation of LC yielded benefits. First, for patients with punctal obstruction, existing examination techniques cannot identify the blind extremity of LC or provide information that aids in punctual incision, including the location, depth of the location, and depth of the blind extremity of LC. In contrast, OCTD could enable LC visualisation even without the use of a contrast agent, thus exhibiting its usefulness for lacrimal surgeries in cases of punctal obstruction. With regard to canalicular obstruction, the source of obstruction was confirmed by direct visualisation of the occluded lumen with the use of a contrast agent (rebamipide suspension). Patients with NLDO, as expected, showed no apparent abnormality in LC morphology and function on the OCTD images. Confirming the integrity of LC via direct visualisation is necessary for the diagnosis of NLDO and to rule out possible LC obstruction. Further studies with larger samples are warranted to determine the potential clinical relevance of these findings.

Our study had several limitations. First, OCTD images were obtained after eversion of the eyelid, which could have distorted LC and altered its original shape. Nonetheless, structural information on LC in an everted eyelid is still useful because several lacrimal surgeries (e.g., punctal dilation and silicon intubation) are performed with everted lids. Returning the eyelid to its original position during rest periods between image acquisitions minimised the impact of lid eversion on LC function. Second, rebamipide ophthalmic suspension, our contrast agent, is used for the treatment of dry eye disease in the clinical setting and is known to increase tear meniscus measurements^17^. Therefore, it is possible that rebamipide instillation may have affected our T1/2 measurements, and alternative contrast agents should be considered in future studies. Despite these limitations, our study highlights the usefulness of a hyper-reflective substance such as a contrast agent for OCTD and, potentially, other OCT imaging techniques.

In conclusion, our findings suggest that OCTD is a revolutionary tool that can facilitate evaluation of the LC morphology and function in a noninvasive manner and, consequently, further our understanding of lacrimal diseases.

## Methods

### Study subjects

This study was reviewed and approved by the Institutional Review Board/Ethics Committee at Kyoto University Graduate School of Medicine (Kyoto, Japan). All study protocols adhered to the tenets of the Declaration of Helsinki. Written informed consent for both study participation, and publication of identifying information/images in an online open-access publication was obtained from all participants after provision of detailed explanations regarding the nature and possible consequences of study participation. OCTD imaging was performed for healthy volunteers without a history of any ocular, lacrimal, or systemic disease and for patients with various disorders of the lacrimal drainage system who visited the Department of Ophthalmology at Kyoto University Hospital between June 2016 and October 2016. An oculoplastic specialist (M.F.) diagnosed the type of pathology by slit-lamp examinations and probing/irrigation.

### Optical coherence tomography dacryography

#### Custom-made optical coherence tomography system

A custom-made (Systems Engineering, Tokyo, Japan) swept-source OCT system that utilised a swept laser operated at a centre wavelength of 1310 nm (Axsun, Billerica, USA) was used to acquire OCTD images (see Supplementary Fig. S2). A schematic of the OCT system is shown in Supplementary Fig. S3. The final illumination power transmitted into the eye was 14 mW, which was within the safety limits specified by the American National Standards Institute^18^. The axial resolution of the system, determined by the effective sweep range of the laser (96 nm), was 13 µm in air and 9 µm in tissue, assuming a tissue refractive index of 1.42. A cube scan obtained with this system contains 1024 × 400 A-scans (scan area, 7 × 7 mm; axial depth, 5 mm). The A-scan and B-scan rates were 50 and 100 Hz, respectively. Multiple joints that allowed for movement in three planes were added to the bottom of the OCT probe to allow appropriate probe positioning and stabilisation.

#### Image acquisition

**Figure 3:**
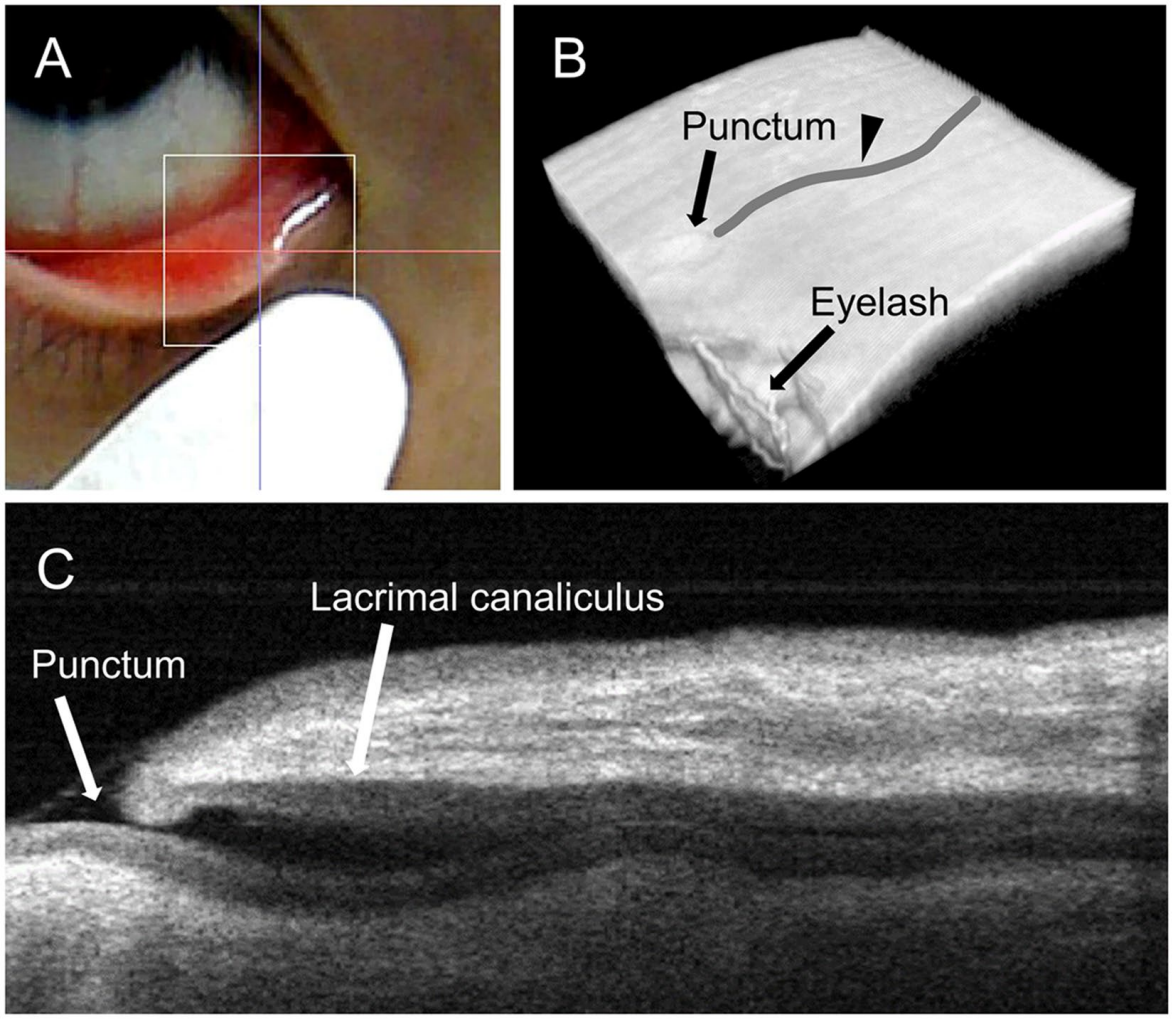
Three-dimensional (3D) optical coherence tomography (OCT) dacryography for visualisation of the lower lacrimal passage in a healthy individual. (**A**) Photograph of a right eye monitored by the CMOS camera. The right inferior lacrimal punctum is the main focus point. 3D OCT imaging of the lower lacrimal passage is performed via the palpebral conjunctiva after eyelid eversion. The OCT scanning area is indicated by the white rectangular area. (**B**) 3D reconstruction of the palpebral conjunctiva and the lacrimal passage within it. (**C**) A B-scan reconstruction created by cube scan reslicing along the curved line shown in (**B**) (arrowhead). The resulting cross-sectional image of the lacrimal canaliculi can be seen.

OCTD provides a greater penetration depth compared with commercial anterior segment OCT (AS-OCT). While AS-OCT can only image a punctum and a part of the vertical LC segment, OCTD can image both the vertical and horizontal LC segments. However, it cannot image the lacrimal drainage system distal to LC. Therefore, in the present study, OCTD was performed for the vertical and horizontal segments of LC. All OCT images were acquired at the right lower eyelid, and all three-dimensional (3D) cube scans of the lower lacrimal passage were obtained from the palpebral conjunctiva after eyelid eversion (Fig. 3). Subjects were instructed to gaze upward to prevent the eyelashes from reflecting the OCT light and producing image artifacts. Subjects were allowed to blink as needed between image acquisitions. Each cube scan was captured within 4 seconds. Rebamipide 2% ophthalmic suspension (Otsuka Pharmaceutical Co., Ltd., Tokyo, Japan)^19^ is a white suspension used to treat dry eye disease. The suspended particles scatter light, and we considered this a suitable property for use of the suspension as a contrast agent for OCTD. All OCTD images were obtained between 1 and 10 minutes after instillation of one drop of rebamipide into the eye.

### Image processing

Raw OCT images were corrected by dividing the incident directional length by the tissue refractive index (1.42). Because LC travels over multiple B-scans in three dimensions, the entire LC length could not be imaged in a single B-scan. Therefore, the acquired cube scans contained an LC reconstruction created by reslicing and reconstructing cube scans using ImageJ software (National Institutes of Health, Bethesda, MD; available at http://rsb.info.nih.gov/ij/index.html), according to a previous study that visualised the post-trabecular aqueous outflow pathway using this technique^20^.

### Morphological and functional evaluations of the lacrimal canaliculus

**Figure 4:**
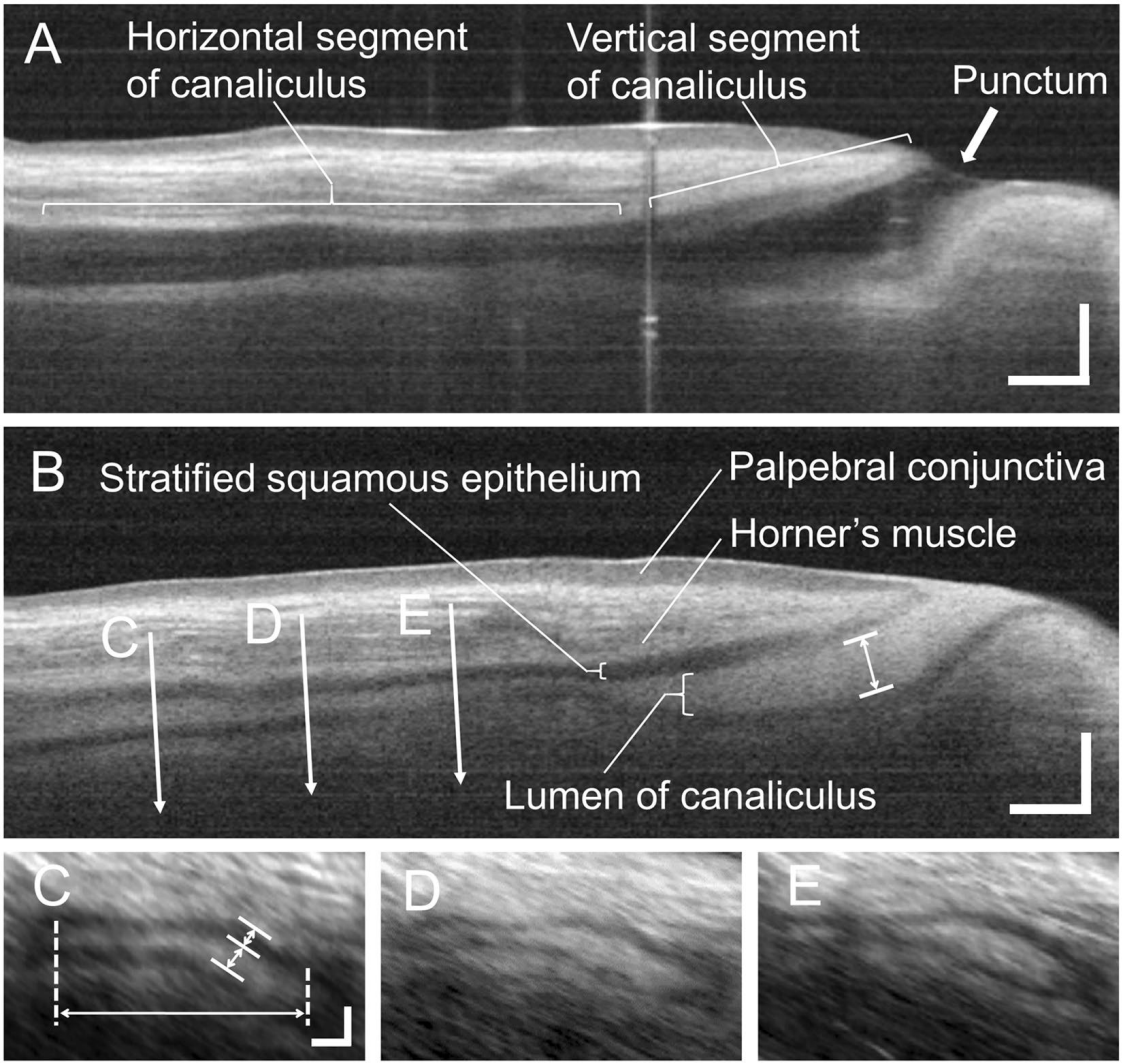
Contrast-enhanced optical coherence tomography dacryography (OCTD) images of the lower lacrimal passage in a healthy individual. (**A**) Longitudinal OCTD image of the lacrimal canaliculus (LC) before contrast agent (rebamipide ocular suspension) instillation. The LC lumen appears as a low-intensity area surrounded by eyelid tissues exhibiting a higher intensity. The LC opening at the punctum is visible. Scale bar = 500 µm. (**B**) Longitudinal OCTD image of LC after ocular contrast agent instillation. The LC lumen appears as a high-intensity area that is easily differentiated from the LC epithelium (narrow bands) between the orbicularis oculi muscle and the LC lumen. Scale bar = 500 µm. (**C**,**D**,**E**) Images of the horizontal LC lumen recorded at 1-mm intervals (arrows) from the proximal horizontal LC indicated in (**B**). LC exhibits a flat lumen, and the transverse length is greater than the anteroposterior length. Scale bar = 200 μm.

OCTD images acquired 1 minute after instillation of the contrast agent were morphologically analysed (Fig. 4). The maximum anteroposterior lumen length in the vertical LC segment was measured on longitudinal OCTD cross-sections oriented along LC. The anteroposterior lumen length, transverse lumen length, lumen area, and epithelium thickness were measured in the horizontal LC segment. All measurements included in the analyses were derived by averaging three individual measurements recorded at intervals of 1 mm along the lumen (Fig. 4). The contrast agent was clearly visible in the LC lumen, enabling extraction of the lumen location in each B-scan and 3D reconstruction using ImageJ software.

Intensity changes in OCTD images obtained every minute for 10 minutes after contrast agent instillation were used to measure LC function. These changes were measured in both the vertical and horizontal segments using ImageJ software. The average grayscale value of a circular region of interest (ROI) measuring >100 µm^2^ in area and including both the horizontal and vertical LC segments was used to measure the image intensity. If a 100-µm^2^ ROI could not be positioned to include the lumen (because of a narrow lumen), multiple circular ROIs were used to examine a region with a total measurement area of >100 µm^2^. To normalise the ROI intensity, the grayscale value for air was defined as the background signal and was subtracted from the lumen grayscale values. The elimination half-life (T1/2) of rebamipide was defined as the time between detection of the maximum intensity and the first instance of a decrease in the intensity to less than half the maximum intensity value.

### Statistical analysis

Data are expressed as means ± standard deviations where applicable. Differences between two groups were assessed using unpaired Student’s t-tests. For comparisons among more than two groups, analysis of variance with post hoc Tukey’s honestly significant difference test was performed. Associations between two groups were analysed using Spearman’s nonparametric correlation analysis. All analyses were performed using StatView software (version 5.0; SAS Institute, Cary, NC). A *P*-value of <0.05 was considered statistically significant.

## Electronic supplementary material

Supplementary Information
