# Supplementary material for: Lacrimal Canaliculus Imaging Using Optical Coherence Tomography Dacryography
Source: Sci Rep. 2018 Jun 28;8:9808. doi: 10.1038/s41598-018-27802-6 (PMC6023928; doi:10.1038/s41598-018-27802-6)
Supplement: Supplementary file 1 — Supplementary Information [file 41598_2018_27802_MOESM1_ESM.pdf]

*Supplementary Information*

# Lacrimal Canaliculus Imaging Using Optical Coherence Tomography Dacryography

Masahiro Fujimoto, Akihito Uji, Ken Ogino, Tadamichi Akagi, Nagahisa Yoshimura

Department of Ophthalmology and Visual Sciences, Kyoto University Graduate School of Medicine, 54 Shogoin Kawahara-cho, Sakyo-ku, Kyoto 606-8507, Japan

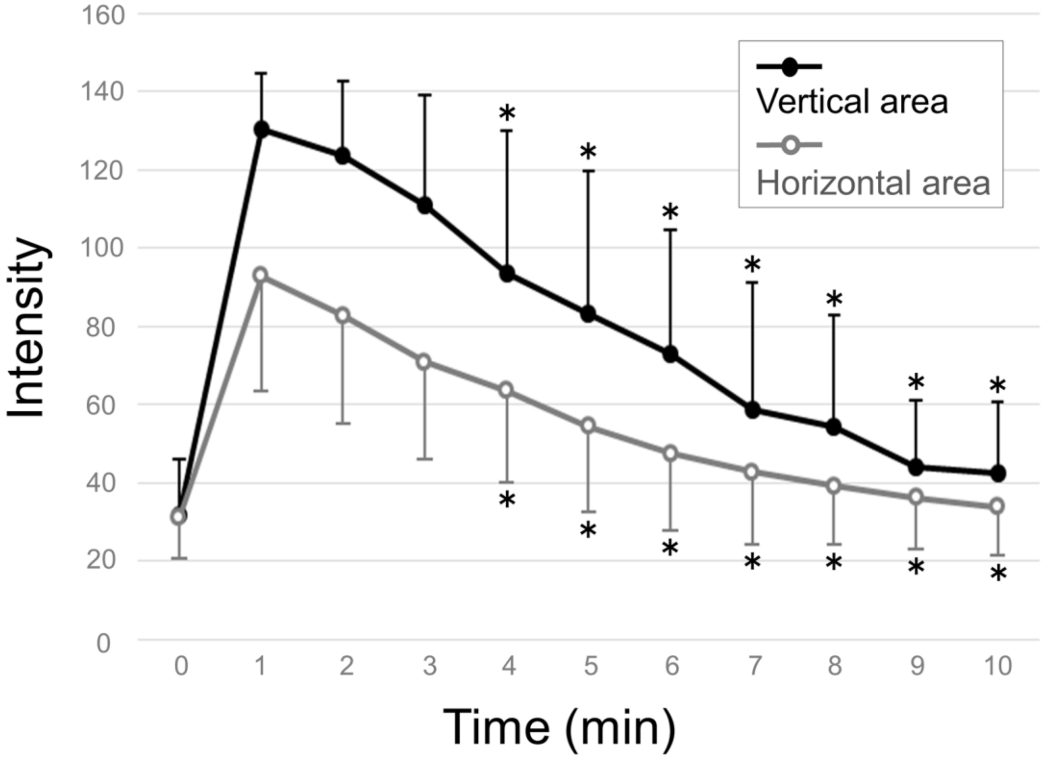


**Supplementary Figure S1. Signal intensity in the lumen of the lacrimal canaliculus (LC) in a healthy individual imaged using contrast-enhanced optical coherence tomography (OCT) dacryography.** The OCT signal intensity shows a peak at 1 minute after contrast agent instillation (rebamipide ophthalmic suspension, time zero) in both the vertical and horizontal LC segments. The signal intensity then gradually decreases and shows a significant difference from the peak value at 4 minutes. *$P$<0.05. Error bars represent one standard deviation.

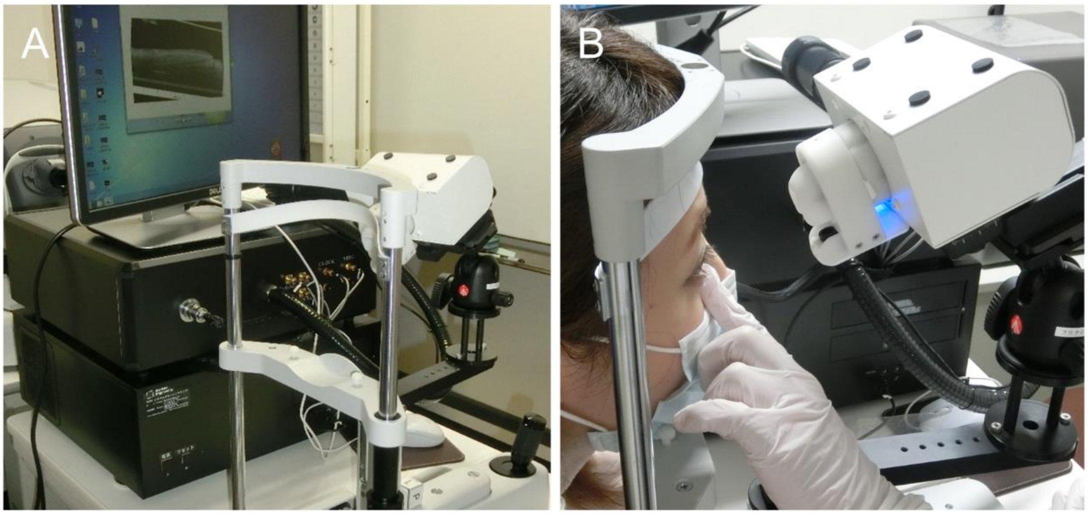


**Supplementary Figure S2. Custom-made swept-source optical coherence tomography (OCT) system used to perform OCT dacryography for assessment of the lacrimal canaliculus.** (A,B) Multiple joints at the bottom allow free movement in three planes to ensure appropriate positioning and stabilization of the OCT probe.

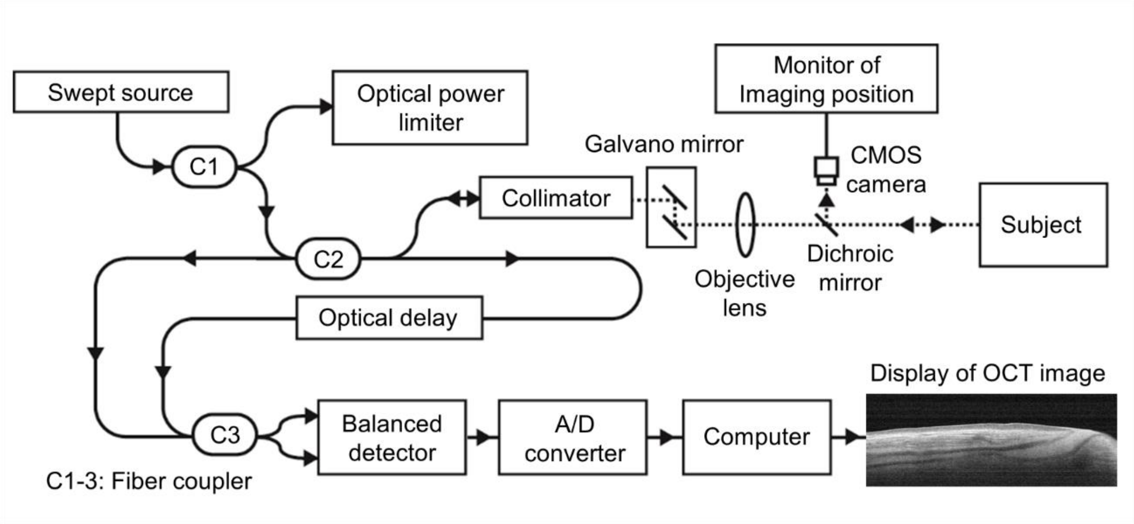


**Supplementary Figure S3. Schematic of the custom-made swept-source optical coherence tomography (OCT) system used to perform OCT dacryography for assessment of the lacrimal canaliculus.** Light from the swept source is split in a 99:1 ratio by a fiber coupler (C1). The 1%fraction of light limits the optical power by turning off the light when it exceeds the normal strength. The 99% fraction of light is split in half by a fiber coupler (C2). One half of the light is collimated, scanned using a Galvano mirror, and used to irradiate the measuring site. The subject is monitored with a CMOS camera. A dichroic mirror reflects visible light and transmits infrared light. Reflected light at the measuring site is interfered with by another light splitter (fiber coupler; C3). Interfering light is perceived by a balanced detector and converted to a digital signal. A cross-sectional image of LC is subsequently created (Computer).
